# Brucellosis in China: history, progress and challenge

**DOI:** 10.1186/s40249-020-00673-8

**Published:** 2020-05-24

**Authors:** Hai Jiang, David O’Callaghan, Jia-Bo Ding

**Affiliations:** 1 State Key Laboratory for Infectious Disease Prevention and Control, Collaborative Innovation Center for Diagnosis and Treatment of Infectious Diseases, National Institute for Communicable Disease Control and Prevention, Chinese Center for Disease Control and Prevention, Beijing, China; 2 VBMI, Universite de Montpellier, INSERM, UFR Medecine, 186, Chemin du Carreau de Lanes, 30908 Nimes Cedex 2, France; 3 Centre National de Reference Brucella, CHU de Nimes, Nimes, France; 4 Department of Diagnostic Technology, China Institute of Veterinary Drug Control, Beijing, China

**Keywords:** Brucellosis, Neglected zoonosis, One health

## Abstract

Brucellosis is a neglected zoonosis. It causes acute febrile illness and a potentially debilitating chronic infection in humans, and livestock infection has substantial socioeconomic impact. Over the past two decades, improvements have been made to better understand the various aspects of human and animal brucellosis. Meanwhile, especially in the developing world, immense challenges that remain in controlling and eradicating brucellosis are novel diagnostics tools and efficacious vaccines. Here, we will focus on the remarkable issues on epidemiological survey, as well as the priority and challenge of brucellosis in China. Brucellosis will be controlled with meaningful collaboration between local and public partnerships effectively applying a One Health framework.

## Background

**Fig. 1:**
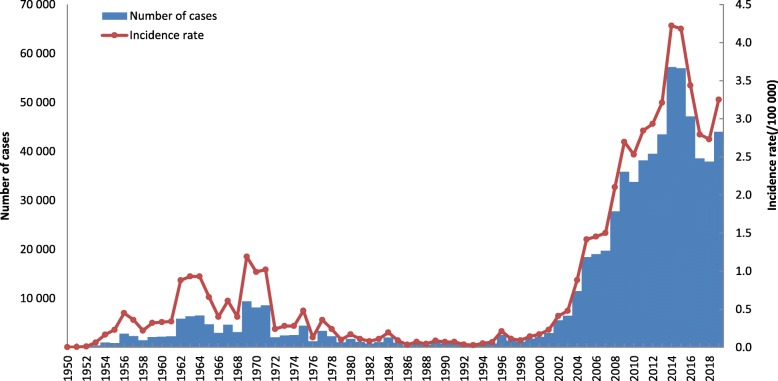
Aggregated number of cases (blue bars) and annual incidence rate (orange line) per 100 000 residents, 1950–2019

Brucellosis remains a serious public health issue, and more efforts are needed to reach the goal of controlling human and animal brucellosis in China. For human brucellosis, a total of 12.5 million persons were examined from 1950 to 1990 with 444 900 sero-positives (positive rate: 3.56%) [1]. In 1993, there were 326 brucellosis cases, with the incidence of 0.028/100 000, which was the lowest in the history. However, the incidence continued to increase since 1995, and brucellosis expanded to all provinces. In 2019, there were 44 036 cases, with the incidence of 3.2513/100 000 (Fig. 1). This may be due to the dynamic growth of animal husbandry in China, which enhances the chance of human infection. In order to grasp the epidemic situation and develop measures for the prevention and control of brucellosis, activities for prevention and control of brucellosis have been gradually introduced. Vaccination for animals and humans was implemented as the main control measure during 1964–1976 in regions with severe epidemics, such as Inner Mongolia, Xinjiang, Qinghai, Ningxia, and Henan provinces [1]. During 1977–1988, a national program for brucellosis control was conducted with the introduction of diagnostic criteria, treatment protocols, and control measures, and vaccination of domestic animals was used as the main control measure. National sentinel surveillance was established in 1990 to monitor the sero-prevalence of brucellosis in humans and animals [2]. Over the past two decades, improvements have been made to better understand the various aspects of humans and animals brucellosis in China. However to control the high endemic will entail enormous challenges, as it has been highlighted by the active discussion around brucellosis intervention strategies in this paper.

## History and evolution of brucellosis in China

Brucellosis incidence in China is divided into three stages: high incidence (1950s–1960s), decline (1970s–1980s), and re-emergence (1990s–) [3]. At the re-emergence stage, brucellosis incidence grew exponentially and spreads to all 32 provinces. During the past decade, outbreaks of human brucellosis have been reported in increasing numbers and with an apparent geographic expansion from the historically affected north of China to southern provinces because of the increasing movement of humans, animals, and animal food products from brucellosis-endemic regions [4].

## Remarkable issues on epidemiological survey of brucellosis

The disease is transmitted to humans mainly by contact with infected animals and the ingestion of infected meat or unpasteurized dairy products [5]. According to the annual brucellosis surveillance report, there were 56 public health emergencies of brucellosis in 2019. These events were mainly related to animal husbandry (33), raw milk (8), or processing and marketing of animal products (8). In 2017, the human brucellosis outbreak (122 cases in Hezhou, Guangxi) was caused by drinking unpasteurized ewe’s milk. Foodborne transmission was a serious risk factor [6–8]. Although abortion in ruminants is a classical symptom of brucellosis, it is only in recent years that it has been fully recognized that brucellosis in pregnant women is associated with obstetric complications [9]. In 2019, we first reported a case of likely mother-to-child transmission of *Brucella* in Hunan Province, China [10]. Although these events are rare, this report highlighted the importance of improving clinical awareness. Brucellosis should be considered for pregnant women presenting with fever, fatigue, and joint-muscle pain not associated with other infections.

In 2019, we described a case to raise the brucellosis clinical diagnosis awareness among clinicians [11]. Brucellosis concomitant with HIV infection are rarely reported. We suggest that there is a need of a prospective investigation on the incidence of brucellosis in HIV patients in the provinces with high incidence of AIDS and brucellosis.

## Genomic-based surveillance of brucellosis

The genus *Brucella* is considered as the most important zoonotic pathogens. Over the last decade, a vast number of *Brucella* genomes have been sequenced allowing us to decipher the evolutionary path of the genus from a soil bacterium to a stealth pathogen. The genus is composed of two groups of strains; the classical *Brucella* including the historical (usually zoonotic) species (*B. melitensis, B. abortus, B. suis. B. ovis, B. canis and B. neotomae)* as well as more recently isolated species (*B. ceti, B. pinnepedialis, B. microti* and *B. papionis*) and a group of atypical strains, which are much older in evolutionary terms. The classical strains are highly conserved at the DNA sequence level, however multilocus sequence typing (MLST) has been used to follow *Brucella* evolution and multiple loci VNTR Analysis (MLVA) has offered the possibilities to follow trace strains at the outbreak level. With the reduced costs and increased speed of next generation sequencing (NGS) technology, new typing methods are being developed to directly exploit whole-genome shotgun (WGS) data.

## Clinical diagnosis

According to the World Health Organization (WHO) factsheet, although approximately 500 000 brucellosis cases are reported annually, the true incidence is always much higher than the reported number of cases. Up to now, there is no distinct and clear guideline for brucellosis diagnosis, with different countries having their own rules. Serologic tests play a fundamental role in the diagnosis of this disease. All of the standard serological tests are based on the detection of antibodies recognizing the O antigen of the *Brucella* lipopolysaccharide (LPS). The interpretation of these tests is usually difficult, particularly in patients with chronic brucellosis, reinfection, and relapse states and in endemic areas where a high frequency of positive serology are observed. Neither the US Center for Disease Control (CDC) nor the WHO offers a specific definition for chronic brucellosis [12, 13]. Lateral flow assays do not require extensive laboratory infrastructure or technical expertise, and compared to the standard of serum tube agglutination (SAT) and/or culture, the sensitivity and specificity were 92–95 and 97%, respectively, in endemic settings [14]. They are, however, very expensive compared to Rose Bengal test (RBT) and SAT. BrucellaCapt (Vircel) is also reported to detect the IgA response that characterizes chronic brucellosis.

The gold standard for diagnosis of brucellosis is a positive culture. Manipulation of *Brucella* presents a risk if the manipulator is untrained or not working with the appropriate confinement (in a microbiological safety cabinet) and unnecessary manipulation (e.g. slide agglutination, antibiotic sensitivity testing) should be avoided. MALDI-TOF-MS is used in developed provincial CDCs and is becoming the method of choice for bacterial identification in some modern diagnostic laboratory. A new spectral database has been developed for the bioMerieux Vitek system which allows the identification of *Brucella* at the species level [15].

Detection of *Brucella* DNA in clinical samples by PCR is a powerful tool when culture is not conclusive. Many assays are based on detection of the *Brucella* specific *IS*711 sequence, which is found in multiple copies in the genomes of all *Brucella.* PCR was effectively employed to rapidly detect *Brucella* DNA in the blood of six suspected cases which all subsequently met confirmed case definitions, and multiplex assays can expedite the confirmation and speciation of *Brucella* isolated by culture and rapidly identify the species and biovar [16].

The atypical strains present a major problem for the diagnostic laboratory. Phenotypically, many do not look like *Brucella* (rapid growth, motile, abnormal metabolic profiles) and many do not produce the persoamine based O antigen meaning that they will not be agglutinated by typing serum, and serological reactions to standard tests based on *Brucella* LPS will be negative.

The identification of new diagnostic and prognostic biomarkers is a major challenge for future research. Since serum and plasma are accessed with relative ease, circulating biomarkers are promising targets. Fine analysis of cytokine levels may give clues. Studies increasingly indicate that dysregulated microRNAs (miRNAs) are associated with bacterial infection. Nevertheless, little is known about miRNAs that respond to *Brucella* infection and their potential clinical value. Although there are many studies on *Brucella* infection, few studies have been devoted to exploring the effects of brucellosis on serum miRNA expression, however miR-103b has been highlighted. Further exploration and validation are required to evaluate the potential target genes of miR-103b and their relationship with the occurrence and development of brucellosis [17].

## Vaccination against brucellosis

As a zoonotic infection, the best way to control human brucellosis is to control the disease in animals. Two vaccines, *B. melitensis* Rev1 for small ruminants and *B. abortus* S19 for bovine brucellosis have been used to control brucellosis throughout the world for many years. Although not perfect, they are the best available options and should be the basis of control with strict compliance to the International Epizootic Office (OIE) guidelines. However, should vaccines be developed to protect the animals or humans, even if doing so may have a negative economic effect on farmers? In cases where a vaccine has been successfully developed, how can we use it in the field without hampering serological diagnosis?

*B. abortus* 104 M has been used as a vaccine against brucellosis in China since 1965. This strain is poorly characterized, is not widely available and concerns exist about its safety. Much more work is required to develop a safe and effective vaccine for humans brucellosis. We can also ask whether such a vaccine is necessary; controlling animals disease and improved food safety (especially avoiding the consumptions of unpasteurized milk products) should be the first line of control [8].

## French-Chinese collaborations

A twinning project was established between the EU Reference Laboratory for Brucellosis (ANSES, Maisons Alfort, France) and the Chinese Animal Health and Epidemiology Centre (CAHEC, located in Qingdao, China) to strengthen the capacities of the CAHEC Brucellosis laboratory. This led to a series of training workshops organized in France by the ANSES laboratory.

Surveillance of humans brucellosis is implemented by the State Key Laboratory for Infectious Disease Prevention and Control in China. There are close contacts with the brucellosis group from the French National Reference Centre in Nimes, with regular visits and discussion of joint projects. We are working on establishing a formal collaboration.

## One health strategies in China

A cornerstone of zoonotic infectious disease epidemiology is the One Health concept. This disease will not be controlled or eradicated without meaningful collaboration between local and public partnerships. The National Brucellosis Control Program (2016–2020) was issued by Ministry of Agriculture and National Health and Family Planning Commission. The OIE Reference Laboratory for Brucellosis (by Dr. Jia-Bo Ding) was established by China Institute of Veterinary Drug Control (CIVDC) in 2019. A series of training workshops will be held every year by China CDC and CIVDC.

## Challenge of future research

Immense challenges of future research are: (1) to develop and validate novel diagnostics to replace blood culture; (2) to develop efficacious animals vaccines that provide better protection to animal and professional populations (3) to develop genomic-based typing method in outbreak detection, source tracing, transmission mode discovery, and new epidemic clone identification.

## Conclusions

Human brucellosis is an easily neglected public health issues in China, which would profit from improved vaccines and simple, specific, and inexpensive diagnostic tests. The disease will be controlled with meaningful collaboration between local and public partnerships effectively applying a One Health framework. We recommended the following preparedness measures that should be taken by all stakeholders in China including (i) strengthening information dissemination and health education on brucellosis; (ii) improving veterinary and public health surveillance, such as preparing the fast detection tests for screening of suspected livestock and products, and setting standard operating procedures for risk assessment.

## Data Availability

Not applicable.

## Abbreviations

MLST: Multilocus sequence typing
MLVA: Multiple-locus variable number tandem repeat analysis
NGS: Next generation sequencing
WGS: Whole-genome shotgun
WHO: World Health Organization
LPS: Lipopolysaccharide
CDC: Center for Disease Control
SAT: Serum tube agglutination
RBT: Rose Bengal test
OIE: International Epizootic Office
CAHEC: Chinese Animal Health and Epidemiology Centre
CIVDC: China Institute of Veterinary Drug Control
